# Long-term cognitive functioning is impaired in ICU-treated COVID-19 patients: a comprehensive controlled neuropsychological study

**DOI:** 10.1186/s13054-022-04092-z

**Published:** 2022-07-20

**Authors:** Henriikka Ollila, Riikka Pihlaja, Sanna Koskinen, Annamari Tuulio-Henriksson, Viljami Salmela, Marjaana Tiainen, Laura Hokkanen, Johanna Hästbacka

**Affiliations:** 1 Department of Anaesthesiology, Intensive Care and Pain Medicine, Helsinki University Hospital and University of Helsinki, Helsinki, Finland; 2 Department of Psychology and Logopaedics, Faculty of Medicine, University of Helsinki, Helsinki, Finland; 3 Division of Neuropsychology, HUS Neurocentre, Helsinki University Hospital and University of Helsinki, Helsinki, Finland; 4 Department of Neurology, Helsinki University Hospital and University of Helsinki, Helsinki, Finland

**Keywords:** COVID-19, Cognitive functioning, Cognitive impairment, Intensive care unit, Long-term outcome

## Abstract

**Background:**

Cognitive impairment has emerged as a common post-acute sequela of coronavirus disease 2019 (COVID-19). We hypothesised that cognitive impairment exists in patients after COVID-19 and that it is most severe in patients admitted to the intensive care unit (ICU).

**Methods:**

This prospective controlled cohort study of 213 participants performed at the Helsinki University Hospital and the University of Helsinki, Finland, comprised three groups of patients—ICU-treated (*n* = 72), ward-treated (*n* = 49), and home-isolated (*n* = 44)—with confirmed COVID-19 between March 13 and December 31, 2020, participating in a comprehensive neuropsychological evaluation six months after the acute phase. Our study included a control group with no history of COVID-19 (*n* = 48). Medical and demographic data were collected from electronic patient records and interviews carried out four months after the acute phase. Questionnaires filled six months after the acute phase provided information about change in cognitive functioning observed by a close informant, as well as the presence of self-reported depressive and post-traumatic symptoms.

**Results:**

The groups differed (effect size *η*^2^_*p*_ = 0.065, *p* = 0.004) in the total cognitive score, calculated from neuropsychological measures in three domains (attention, executive functions, and memory). Both ICU-treated (*p* = 0.011) and ward-treated patients (*p* = 0.005) performed worse than home-isolated patients. Among those with more than 12 years of education, ICU-treated patients performed worse in the attention domain than ward-treated patients (*p* = 0.021) or non-COVID controls (*p* = 0.045); ICU-treated male patients, in particular, were impaired in executive functions (*p* = 0.037).

**Conclusions:**

ICU-treated COVID-19 patients, compared to patients with less severe acute COVID-19 or non-COVID controls, showed more severe long-term cognitive impairment. Among those with more than 12 years of education, impairment existed particularly in the domains of attention and for men, of executive functions.

*Trial registration number*: ClinicalTrials.gov NCT04864938, retrospectively registered February 9, 2021

**Supplementary Information:**

The online version contains supplementary material available at 10.1186/s13054-022-04092-z.

## Background

Long-term cognitive impairment is a common complication of coronavirus disease 2019 (COVID-19) caused by severe acute respiratory syndrome coronavirus 2 (SARS-CoV-2) [1]. The pathway causing neurological complications in COVID-19 is still unclear, and several theories concerning the mechanisms exist: direct neuroinvasion via a neuronal or haematogenous route, blood–brain barrier disruption due to cytokine storm and hypoxaemia, thrombotic vascular events, and autoimmunity, or their combination [2–4].

Survivors of acute respiratory distress syndrome (ARDS), a potential complication also in COVID-19, have a high prevalence (46–80%) of long-term cognitive impairment [5]. Twelve months after critical illness, a significant number of critical care survivors treated in general intensive care units (ICUs) suffer from cognitive impairment comparable to moderate traumatic brain injury (34%) or mild Alzheimer’s disease (24%); this impairment was also observable in patients under 50 years of age [6]. In a recent meta-analysis of 43 studies that included patients assessed 12 or more weeks after COVID-19, cognitive impairment existed in 22% [1].

After the previous coronavirus epidemics of severe acute respiratory syndrome (SARS) and Middle East respiratory syndrome (MERS), subjective cognitive dysfunction followed in sufferers. Of SARS patients, 27% reported subjective difficulties in concentration and 43% in memory after a mean follow-up of 42 days [7]. Of SARS and MERS patients studied over a follow-up period of six weeks to 39 months, more than 15% suffered from long-term deficits of concentration and memory, and fatigue [8]. During mean follow-up time of 35 months, 23% were unable to return to work [8].

The incidence of reported cognitive impairment after COVID-19 has been very variable (15–100%) when assessed with the Montreal Cognitive Assessment or Telephone Interview for Cognitive Status (normal or modified version) [9–14]. Deficits emerged, especially in executive, visuospatial, concentration and language, short-term memory, and attention tasks [9, 13, 14]. Acute disease severity has had varying effects on cognitive functioning [11, 12]. In a review focusing on objective neuropsychological testing on COVID-19 patients, global cognitive impairment occurred in 15–80% of individuals. Seven out of 12 studies focusing on attention and executive functions discovered some difficulties in those domains. In three out of four studies, memory impairment existed [15].

Many studies have depended upon questionnaires, telephone interviews or Internet testing, without performing objective comprehensive neuropsychological assessment in person, and most studies lack a non-COVID control group, despite the impact of the pandemic on the whole of society. We designed the current study to explore, following an objective, comprehensive testing method, whether the long-term cognitive functioning is associated with the maximum level of care needed in the acute phase of COVID-19; this was done while also accounting for confounders and assessing the eventual effects on the population without a history of COVID-19 but affected by the societal impact of the pandemic. To find potential confounding factors, we measured self-reported depressive and post-traumatic stress symptoms, and informant-reported change in cognitive status; we also evaluated functional outcome. We hypothesised that cognitive impairment exists in patients after COVID-19 and that it is most severe in patients admitted to the ICU.

## Methods

The Recovery after a critical COVID-19 infection (RECOVID) study project is registered at ClinicalTrials.gov (NCT04864938). The ethics board of Helsinki University Hospital approved the study protocol (HUS-1949-2020). All subjects gave written informed consent to the study and were treated according to the principles of the Declaration of Helsinki.

### Patients

Between 12 March 2020 and 31 December 2020, we recruited adults aged 18 years or older with a confirmed (reverse transcription-polymerase chain reaction or antibody testing) SARS-CoV-2 infection at Helsinki University Hospital. Due to the language sensitivity of the testing, only subjects fluent in Finnish were eligible. Only patients with complete neuropsychological assessment data were included in the present study. A control group with no history of SARS-CoV-2 infection was also recruited.

Exclusion criteria were prior major neurological diagnosis (traumatic brain injury, dementia, stroke, Parkinson’s disease), developmental disability, or substantially impaired hearing or vision. We sent a written invitation to all consecutive eligible ICU survivors within three months of hospital discharge, invited them to a follow-up clinic, and asked them to participate in the study (ICU group). Hospitalised non-ICU COVID-19 patients (WARD group) were recruited during the index hospitalisation or at a follow-up pulmonology clinic. The home-isolated subjects (HOME group) and non-COVID controls (CONTROL group) were recruited by media announcements.

### Data collection

We collected data on the hospitalised cohorts from electronic patient records and intensive care patient data management systems (Apotti, Epic™, Verona, USA; PICIS™, Wakefield, USA; Uranus™, CGI, Montreal, Canada); data on the home-isolated group and the non-COVID controls were collected from the patient records and the study subjects by telephone interviews. We recorded age, sex, years of education, comorbidities, body mass index (BMI), Charlson comorbidity index (CCI) [16], and admission data (see Additional file 1 for definitions of the clinical variables).

### Cognitive functioning

The primary outcome measure of this study was cognitive functioning six months after hospital discharge, evaluated via a comprehensive neuropsychological assessment. The control group underwent an identical evaluation during the spring of 2021. We selected the main outcome variables to represent three domains: memory, executive functions, and attention. We evaluated attention with Wechsler Adult Intelligence Scale-IV coding [17], Continuous Performance Test [18, 19], and Stroop Naming [20], executive functions with Trail Making B [21], Stroop Interference [20], and Frontal Assessment Battery [22], and memory with Wechsler Memory Scale version III (WMS-III) word list, delayed recall, WMS-III logical memory, delayed recall [23], and Rey Complex Figure, delayed recall [24]. The outcome variables were standardised to Z-scores (see Additional file 1: Table E1 for more details on neuropsychological methods), and we calculated a total cognitive score as the sum of the domain scores. In all scores, a higher value indicates a better performance.

### Functional and psychological outcomes

A telephone interview conducted at four months included a structured list of questions on symptoms, assessment of general functional outcome using a modified Rankin scale (mRS, Additional file 1: Table E2) [25], and a record of employment status. Approximately six months after acute illness, the subjects filled out a questionnaire, which included a measure of depression (Patient Health Questionnaire 9, PHQ-9, Additional file 1: Table E3) [26] and post-traumatic stress (Impact of Event Scale-6, IES-6, Additional file 1: Table E4) [27]. In both measures, higher scores indicate more problems in psychological well-being. For each patient, a close informant also completed a questionnaire (modified Informant Questionnaire on Cognitive Decline in the Elderly, IQCODE), evaluating the potential change in the subject’s cognitive status from the time before COVID-19 [28]. Scores of three or below indicate improved or no change in cognition, while scores above three indicate a decline.

### Statistical comparisons

We used a general linear model (GLM) for comparing cognitive functioning in the different groups. We added age as a covariate, and sex and educational level (more than 12 years vs. 12 years or less) as additional fixed factors in univariate and multivariate models. We report partial eta squared (*η*^2^_*p*_) for effect size and Bonferroni corrected *p* values to correct for multiple comparisons in pairwise comparisons. Effect sizes of 0.01 indicate a small, 0.06 a medium, and 0.14 or higher a large effect [29].

A hierarchical linear regression analysis was conducted to examine the association between ICU-related variables and cognitive performance. Background variables (age, sex, educational level) were entered first (in the first block), followed by each ICU-related variable (CCI, pronation, duration of invasive mechanical ventilation [IMV-duration] and delirium, see Additional file 1) entered one by one (in the subsequent blocks).

Continuous variables are presented as median and interquartile range (IQR) or mean and standard deviation (SD), while categorical variables are expressed as number of patients (percentage). *P* values < 0.05 were considered statistically significant. The Chi-squared test or Fisher’s exact test was used for categorical variables, and a nonparametric test (Kruskal–Wallis test) was used in the case of a non-normal distribution. Analyses were performed with IBM® SPSS® Statistics (version 27.0.1.0) and RStudio® (version 1.4.1717, PBC, Boston, MA, USA).

## Results

### Patient characteristics

**Fig. 1:**
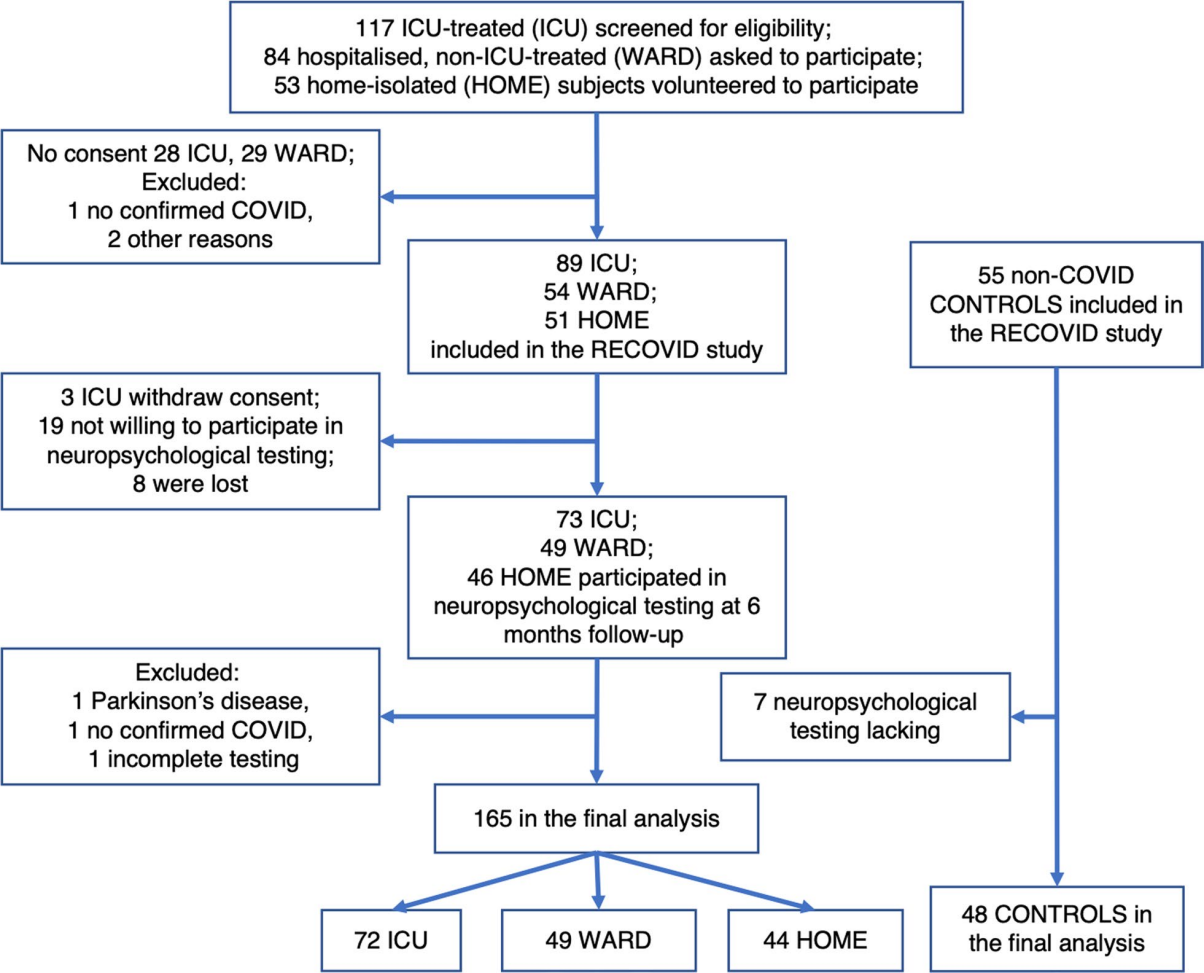
Flow chart showing the number of subjects included in the final analysis

Our study consisted of 165 patients with a history of a confirmed SARS-CoV-2 infection and 48 non-COVID controls. Figure 1 shows the study flow chart. Table 1 shows the patient and treatment characteristics, employment status, and general outcome measures. In the ICU group, 46 (64%) required invasive mechanical ventilation (IMV), and of those patients, 28 (64%) had the lowest PaO_2_/FiO_2_-ratio less than 100 mmHg indicating severe ARDS; nearly 50% of IMV-patients received at least one prone position treatment (Table 2). None of the patients was treated with extracorporeal membrane oxygenation. One in three patients in the ICU group were delirious, compared to none in the WARD group.

**Table 1:** Patient characteristics and general outcome

	ICU *n* = 72	WARD *n* = 49	HOME *n* = 44	CONTROL *n* = 48	*p* value
Age, years, median (IQR)	59 (49–65.3)	57 (49–62)	44.5 (34.3–52)	56 (49–63.3)	< 0.001
Sex, male, *n* (%)	44 (61.1)	18 (36.7)	12 (26.7)	25 (52.1)	0.002
*Education*					
Years, mean (SD)	13.6 (2.7)	14.9 (2.7)	15.6 (2.1)	15.4 (2.9)	< 0.001
> 12 years, *n* (%)	44 (61)	38 (78)	38 (86)	39 (81)	0.009
*Comorbidities*					
Hypertension, *n* (%)	39 (54.2)	14 (28.6)	8 (18.2)	11 (22.9)	< 0.001
Hypercholesterolaemia, *n* (%)	24 (33.3)	10 (20.4)	4 (9.1)	7 (14.6)	0.009
Heart disease, *n* (%)	12 (16.7)	3 (6.1)	4 (9.1)	1 (2.1)	0.05
Diabetes, *n* (%)	16 (22.2)	5 (10.2)	2 (4.5)	1 (2.1)	0.002
Malignancies, *n* (%)	5 (6.9)	2 (4.1)	2 (4.5)	1 (2.1)	NS
Asthma, *n* (%)	12 (16.7)	13 (26.5)	4 (9.1)	2 (4.2)	0.01
COPD, *n* (%)	0 (0)	3 (6.1)	0	0	0.03
Kidney disease, *n* (%)	3 (4.2)	0 (0)	0	0	NS
Liver disease, *n* (%)	0 (0)	2 (4.1)	1 (2.3)	0	NS
Psychiatric or neurological comorbidity, *n* (%)	5 (6.9)	4 (8.2)	1 (2.3)	5 (10.4)	NS
BMI kg/m^2^, median (IQR)	30.1 (26.9–34.3)^a^	28.6 (26.0–33.5)^b^	24.9 (23.5–27.6)^c^	–	< 0.001
CCI, median (IQR)	2 (0.5–3.0)	1 (1.0–3.0)	–	–	NS
*Admission data*					
Length of hospital stay, days, median (IQR)	20 (15–26)	8 (5–11)	–	–	< 0.001
Length of ICU stay, days, median (IQR)	11 (5.8–17.3)	–	–	–	
Supplementary oxygen, days, median (IQR)	19 (14–23)	6 (1–9)	–	–	< 0.001
*Outcome*					
mRS^d^ at 4 months 0–1, *n* (%)	39 (54.9)	28 (63.6)	29 (69.0)	48 (100)	< 0.001
2, *n* (%)	29 (40.8)	15 (34.1)	13 (31.0)	–	
3–4, *n* (%)	3 (4.2)	1 (2.3)	–	–	
Employed prior, *n* (%)	45 (62.5)	34 (69.4)	34 (77.3)	37 (77.1)	NS
Full return to work at 4 months, *n* (%)	27 (60)	26 (76.5)	31 (91.2)	–	< 0.001
IQCODE^e^ > 3, *n* (%)	28 (50.9)	13 (31.0)	10 (30.3)	6 (15.0)	0.003
IES-6^f^ at 6 months, mean (SD)	0.97 (0.81)	0.99 (0.82)	0.88 (0.79)	0.52 (0.55)	0.011
PHQ-9^f^ at 6 months, mean (SD)	4.7 (5.0)	5.1 (4.4)	5.2 (4.7)	2.1 (2.0)	0.001

*IQR* Interquartile range*, SD* standard deviation, *NS* non-significant, *COPD* chronic obstructive pulmonary disease, *BMI* body mass index, *CCI* Charlson comorbidity index, *mRS* modified Rankin scale (measure of functional outcome), *IQCODE* Informant Questionnaire on Cognitive Decline in the Elderly, *IES-6* Impact of Event Scale-6 (measure of post-traumatic stress), *PHQ-9* Patient Health Questionnaire 9 (measure of depression)

^a^Data available for 70 of 72 study subjects

^b^Data available for 38 of 49 study subjects

^c^Data available for 25 of 44 study subjects

^d^Data available for 205 of 213 study subjects

^e^Data available for 170 of 213 study subjects

^f^Data available for 186 of 213 study subjects

**Table 2:** Features of ICU-treated patients (*n* = 72)

ICU variable	Result
IMV, *n* (%)	46 (63.9)
IMV, days, median (IQR)	13 (8.3–16.8)
Lowest PaO_2_/FiO_2_ < 100 mmHg, *n* (%)^a^	28 (63.6)
Lowest PaO_2_/FiO_2_ 100–149 mmHg, *n* (%)^a^	15 (34.1)
Lowest PaO_2_/FiO_2_ 150–199 mmHg, *n* (%)^a^	1 (2.3)
Tracheostomy, *n* (%)	7 (15.2)
Pronation, *n* (% of IMV)	22 (47.8)
Number of proning episodes, median (IQR)	2 (1–3)
Delirium diagnosed during ICU stay, *n* (%)^b^	24 (33.3)
Length of delirium, days, median (IQR)	2 (1–3)
AKI diagnosed during ICU stay, *n* (%)	11 (15.3)
RRT in ICU, *n* (%)	6 (8.3)
Days RRT given in ICU, median (IQR)^c^	14 (13–20)
SAPS 24 h, median (IQR)^d^	27 (20–37)
APACHE 24 h, median (IQR)^d^	18 (13.5–20.5)
SOFA 24 h, median (IQR)^d^	5 (3–8)

*ICU* Intensive care unit, *IMV* invasive mechanical ventilation, *PaO*_2_*/FiO*_2_ ratio of arterial oxygen partial pressure to fractional inspired oxygen, *AKI* acute kidney injury, *RRT* renal replacement therapy, *SAPS* Simplified Acute Physiology Score during the first 24 h of ICU stay, *APACHE* Acute Physiology and Chronic Health Evaluation during the first 24 h of ICU stay, *SOFA* Sequential Organ Failure Assessment during the first 24 h of ICU stay

^a^PaO_2_/FiO_2_ ratio unavailable for 2 patients treated in another region

^b^Delirium diagnosed as ICDSC 4 or more points in 21 of 70 patients and clinically in 3 of 70 patients. Data unavailable for 2 patients

^c^Data available for 5 of 6 patients. 1 patient was RRT dependent at ICU discharge

^d^Data available for 59 of 72 patients

### Cognitive performance

Comprehensive neuropsychological testing occurred on average 209 days (SD 25) after hospital admission of the ICU and WARD patients or positive COVID-19 test result of the non-hospitalised HOME patients; for patients admitted to the hospital, this testing was approximately six months after discharge.

#### Total cognitive score

**Fig. 2:**
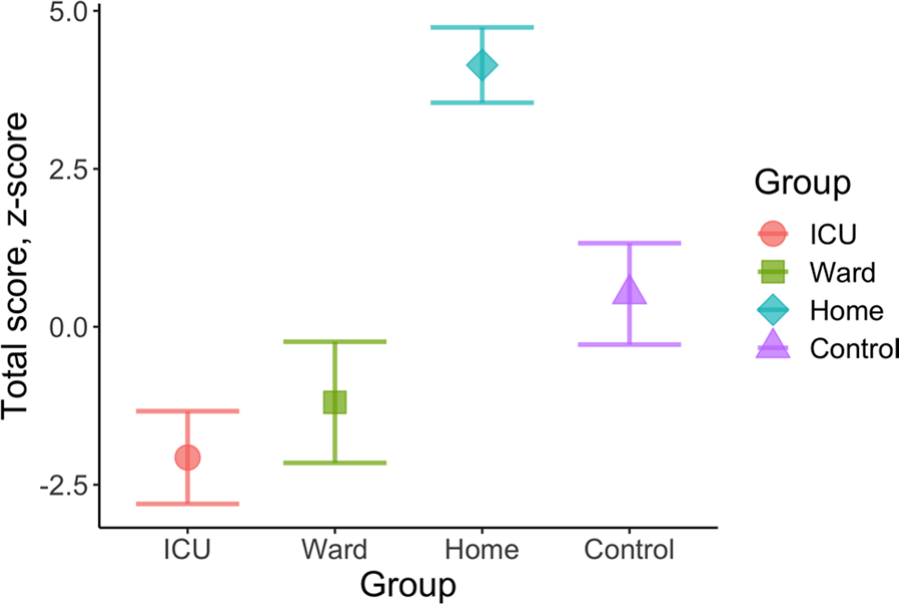
The total cognitive score in the three patient groups and the control group. Data presented as means and standard errors of the mean. Statistically significant difference in pairwise comparisons ICU < HOME (*p* = 0.011) and WARD < HOME (*p* = 0.005)

Figure 2 shows the total cognitive score in the three patient groups (ICU, WARD, and HOME) and the CONTROL group. In GLM analysis (Table 3), the total cognitive score at six months post-COVID differed between the groups (*F* = 4.506, *p* = 0.004, *η*^2^_*p*_ = 0.065). In pairwise comparisons, both ICU (*p* = 0.011) and WARD patients (*p* = 0.005) performed worse than HOME group. The effects of age (*p* < 0.001) and educational level (*p* < 0.001) were also significant, and a significant 2-way interaction was observed between group and education (*p* = 0.003) as well as a significant 3-way interaction between group and sex and education (*p* = 0.038). When the measure of depression and the measure of post-traumatic stress were added into the GLM, the effect size for the group difference increased (*F* = 5.759, *p* < 0.001, *η*^2^_*p*_ = 0.094).

**Table 3:** Means and standard deviations (SD) of the cognitive scores in the study groups

	N	ICU	WARD	HOME	CONTROL	ES of group	ES of age	ES of education	ES of sex	Significant interactions	Significant pairwise differences
Total score mean (SD)	213	− 2.342 (6.526)	− 0.719 (6.274)	4.095 (3.595)	0.520 (5.560)	.065 **	.292 ***	.150 ***	.000	Group and education, group and sex and education	ICU < HOME, WARD < HOME
Domain scores multivariate model	159^a^					.039 *	.319 ***	–	.020		
Attention, mean (SD)	159^a^	− 0.342 (2.387)	0.690 (1.665)	1.240 (1.378)	0.865 (1.607)	.070 **	.273 ***	–	.000		ICU < WARD, ICU < CONTROL
Executive functions, mean (SD)	159^a^	− 0.185 (2.835)	0.413 (1.789)	1.396 (1.135)	0.880 (1.536)	.036	.247 ***	–	.001	Group and sex	
Memory, mean (SD)	159^a^	− 0.233 (2.201)	0.435 (2.117)	1.861 (1.843)	0.274 (2.011)	.048	.148 ***	–	.018		

Effect sizes (ES) for group, age, education level and sex in univariate and multivariate general linear models, and significant pairwise differences between the study groups

^a^Only cases with > 12 years of education. ES = effect size (partial eta squared): 0.01 denotes a small, 0.06 a medium, and 0.14 or higher a large effect size; *p < 0.05, **p < 0.01, ***p < 0.001

#### Domain-specific scores

**Fig. 3:**
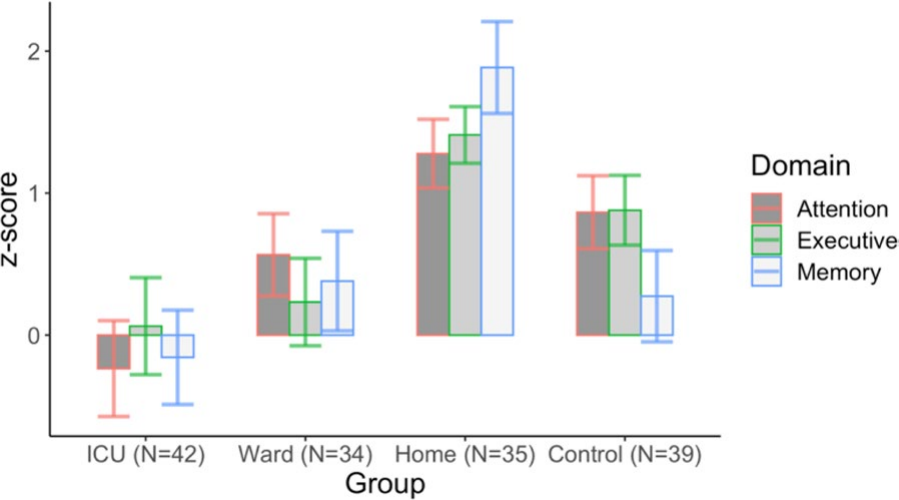
The domain-specific cognitive scores for attention, executive functions, and memory in the group with more than 12 years of education. Data presented as means and standard errors of the mean. Statistically significant difference between the groups, Wilks’ lambda *p* = 0.039

As educational level was a significant confounder in the total score, the domain scores were analysed in the group with more than 12 years of education only (*n* = 159, 75% of total participants) (Fig. 3). The three domain scores in a multivariate model with age and sex included showed a difference between the groups (Wilks’ lambda *p* = 0.039, *η*^2^_*p*_ = 0.039). The effect of age was also significant (Table 3). In attention, the groups differed (*F* = 3.748, *p* = 0.012, *η*^2^_*p*_ = 0.070) and in pairwise comparisons, ICU patients had a worse performance than WARD patients (*p* = 0.021) and controls (*p* = 0.045). No other differences emerged. In executive functions, a significant 2-way interaction between group and sex was found (*F* = 3.613, *p* = 0.015, *η*^2^_*p*_ = 0.067), and the sex differences were analysed in the ICU patients, showing that men (*n* = 23) performed worse than women (*n* = 21) (*F* = 4.654, *p* = 0.037, *η*^2^_*p*_ = 0.102).

#### Comparison of the ICU variables

For ICU patients, we examined the association between the ICU-specific variables and the total cognitive score. Table 4 presents the models, AIC values, and changes in R^2^ value after each added variable. The total percentage of accounted variance for all the included variables was 54.8% (model 5). Background variables (model 1) accounted for 44.8%, and adding CCI in model 2 resulted in a significant increase in the variance explained (6.8%, *p* = 0.003). No increase in the explained variance resulted from adding more variables (models 3–5, proning, IMV-duration, or delirium).

**Table 4:** Hierarchical regression of ICU-specific variables in predicting the total neuropsychological score

	Included variables	*R*	*R*^2^	Adj *R*^2^	AIC	Overall model test		Model comparison		
						*F* (*df*)	*p*	Δ*R*^2^	*F* (*df*)	*p*
1	Age, sex, education	0.670	0.448	0.424	435	18.2 (3.67)	< .001			
2	+ CCI	0.718	0.516	0.487	427	17.6 (4.66)	< .001	0.06771	9.235 (1.66)	**0.003**
3	+ Proning	0.720	0.518	0.481	429	14.0 (5.65)	< .001	0.00212	0.286 (1.65)	0.594
4	+ IMV-duration	0.731	0.535	0.491	429	12.3 (6.64)	< .001	0.01648	2.267 (1.64)	0.137
5	+ Delirium	0.740	0.548	0.498	429	10.9 (7.63)	< .001	0.01365	1.904 (1.63)	0.173

Statistical significance marked in bold

All models include age, sex, and education. Other variables are added one by one in models 2–5. *R*^2^ Coefficient of determination, Adj *R*^2^ Adjusted *R*^2^, *AIC* Akaike information criterion, *df* Degrees of freedom, Δ*R*^2^ Change in *R*^2^, *CCI* Charlson comorbidity index, *IMV* Invasive mechanical ventilation

### Functional and psychological outcome

In a telephone interview conducted on average 151 days (SD 33) after hospital admission or positive COVID-19 test result (home-isolated patients), 60–91% of those employed before the infection had returned to full work, the proportion being lowest in the ICU group (Table 1). Assessment of the mRS at the same time point showed very low incidence (2–4%) of moderate or moderately severe disability (scores 3 or 4); 55–69% of the COVID-cohort were either symptomless or had some minor symptoms, but no disability, and 31–41% had given up some activities, indicating slight disability (Table 1). Questionnaires filled out on average 205 days (SD 26) after hospital admission or positive COVID-19 test result showed higher scores for all COVID-19 patient groups compared to controls, indicating more problems both with post-traumatic stress and depression (*p* = 0.011 and *p* = 0.001, respectively) (Table 1). An IQCODE score of more than three points, indicating a change in a study subject’s cognitive functioning as assessed by a close informant, occurred for 51% of patients in the ICU group, 31% in the WARD group, 30% in the HOME group, and 15% in the CONTROL group (*p* = 0.003) (Table 1). The total cognitive score was associated with the mRS (ANOVA F = 4.719, *p* = 0.003, *η*^2^_*p*_ = 0.067), and the IQCODE (ANOVA *F* = 4.819, *p* = 0.030, *η*^2^_*p*_ = 0.028).

## Discussion

In this prospective controlled cohort study of 213 participants, the total cognitive score consisting of attention, executive functions, and memory domains at six months post-discharge was impaired in patients with COVID-19, and the impairment was the greatest in the more severely ill in the acute phase of COVID-19. The group differences remained when controlling for age, sex, education, depression, and post-traumatic stress. Among patients with more than 12 years of education, the cognitive scores of the ICU-treated patients, when compared to other COVID-19 groups and controls, were significantly lower in attention and, for men, in executive functions. For ICU patients, an association existed between total cognitive score and the CCI. Surprisingly, factors associated with the severity of respiratory dysfunction, such as proning, and duration of IMV, or delirium, showed no such association.

This finding of impairment, particularly in attention and executive functions, is similar to previously reported [9, 15, 30–32]. Some studies have reported female sex as a risk factor for the post-COVID-19 condition defined by World Health Organization [33, 34]. Although cognitive dysfunction is among the common symptoms of the post-COVID-19 condition [35], long-term objective cognitive impairment might be a separate entity and, hence, have different risk factors. Home-isolated participants performed well in neuropsychological assessment, which suggests cognitive impairment after COVID-19 is correlated with the severity of the disease rather than being a direct consequence of the viral infection. Our data support the recent finding that ICU survivors express more long-term cognitive impairment compared to those with less severe acute infection [32].

A meta-analysis of critically ill patients from 2015 discovered delirium in 31.8% [36], which is very close to the incidence of 33.3% in our ICU cohort. A review of COVID patients has, however, reported a markedly higher delirium incidence of over 50% in the general COVID-19 population, and 65 to 80% in those admitted to the ICU, making delirium the most common neurocognitive condition for hospitalised COVID-19 patients [37, 38]. In the studies reviewed, a validated delirium tool was seldom in use [37]. Delirium is associated with increased mortality both in the general ICU population and in COVID-19 patients [36, 39], and our cohort only included those who survived to six-month follow-up, which might explain the difference in delirium incidence. Delirium is also an independent predictor of long-term cognitive impairment [36, 40–42]. During critical illness, pre-existing cognitive impairment predisposes a person to delirium; it is unclear whether this finding partly explains the correlation between delirium during ICU treatment and post-ICU cognitive impairment [43]. Surprisingly, in our study, no association existed between delirium and cognitive dysfunction beyond the association explained by demographics and the CCI. One possible explanation is a selection bias towards those with better functional outcomes. Our study also excluded patients with known pre-existing cognitive decline.

In our cohort of 113 COVID-19 patients in working life before COVID-19, at four months, 84 (74.3%) had returned to full-time work; the proportion was lowest (60%) in the ICU group and highest (91%) in the HOME group. This finding is in line with a large (*N* = 1077) cohort study from the UK, in which, after a follow-up of 5.9 months, 113 (17.8%) of COVID-19 patients were unable to return to work, and 124 (19.3%) had to change their occupational status because of health reasons [11]. In Australia, six months after critical COVID-19, 11% of patients reported being unemployed due to health reasons [44]. A study comparing ICU-treated COVID-19 patients’ employment, health-related quality of life, emotional health, and pain to matched ICU-treated non-COVID patients discovered similar outcomes in both groups; at follow-up, 54% of the previously employed had returned to work and 46% had symptoms of anxiety [45]. In a BRAIN-ICU cohort of respiratory failure and shock patients, at three months follow-up, 62% had reduced employment. At 12 months, this reduction persisted in 49% and was marginally correlated with worse cognitive functioning [46]. Also, in a study of general ICU patients, 55% had returned to work or study at 12 months [47]. The reason for the relatively high employment rate in our cohort at four months is unclear, but one explanation might be that COVID-19 patients more often than general ICU patients had single organ failure even if the lengths of ICU and hospital stays were longer.

The functional outcome assessed with the mRS at four months showed that more than one third of COVID-19 patients had had to abandon some activities that they could perform pre-COVID, indicating slight disability (score 2); however, only a small minority (3 participants in the ICU group and 1 in the WARD group) was classified with a score of 3 or 4, indicating moderate or moderately severe disability. This finding differs considerably from the findings of a study performed in New York with a cohort of 196 hospitalised COVID-19 patients with neurological complications and 186 hospitalised COVID-19 patients without neurological complications. At six months, for 56% in the non-neurological group and 69% in the neurological group, they found an mRS of > 2, indicating moderate or worse disability; 64% and 41%, respectively, had returned to work [48]. An Austrian study followed COVID-19 patients for three months to assess neurological signs and symptoms; the majority had a good functional outcome with a median mRS of 1 [49]. Of Australian COVID-19 ICU survivors assessed at six months, 39% showed a new disability [44], which is in line with our finding that in the ICU group, the mRS indicated some disability in 45% of patients. Our better results in functional outcome compared to those from New York can be partly explained by cohort differences; our patients were younger, more often female, and had fewer comorbidities, and a quarter of them was non-hospitalised.

All groups of COVID-19 patients had more symptoms of depression and post-traumatic stress at six months compared to non-COVID controls; this finding is similar to other studies [9, 50–52]. The mean scores in all study groups remained below the cut-off (1.75) earlier suggested indicative of post-traumatic stress disorder in ARDS patients [27]. Also for depression, the mean scores remained below the level associated with depressive disorders, suggesting predominantly borderline mild depression in the COVID-19 patient groups (Additional file 1: Table E3) [26]. Interestingly, the home-isolated group also experienced post-traumatic stress symptoms at a similar level compared to other COVID-19 patients. Hence, differences in depression or post-traumatic symptoms offered no explanation for the differences seen in neuropsychological assessment. Our finding of non-COVID controls experiencing psychological symptoms and a degree of cognitive impairment during the pandemic highlights the importance of a control group in this field of research.

The strength of our study was that we assessed our participants with a comprehensive neuropsychological test battery six months after hospital discharge. In addition, our sample size was reasonable, as shown by the observed medium-sized effect sizes of our results, and we included participants with different disease severities and a non-COVID control group. With the control group, we were able to account for the potential confounding factors of age, sex, educational background, and living in the same region.

Some limitations do exist. First, the groups differed in their compositions, as ICU-treated patients were older, more often men, and had more comorbidities, whereas the home-isolated participants were younger, more often women, and had more years of education. The participant compositions of the WARD and CONTROL groups fell in between those of the ICU and HOME groups. This fact, although reflecting the risk factors for developing severe acute disease, may have influenced our findings despite the included statistical covariates, because neuropsychological performance six months after the infection is dependent on the baseline cognitive level. Moreover, the performance is affected by age and educational background as seen in the large effect sizes of these variables. This likely explained the high cognitive performance of the HOME group seen in the table and figures, which show the unadjusted values. Second, the baseline cognitive level of the participants was unknown: thus, some individuals may have performed worse than before, even if their post-COVID-19 level was similar to the controls. No validated measure for pre-morbid cognitive performance level is available in the Finnish language. However, known cognitive decline was an exclusion criterion to the study. Moreover, a close informant answered the IQCODE questionnaire to compare a patient’s current cognitive performance to that in the pre-morbid situation. The results showed that the close informants detected cognitive decline, which supports the interpretation of a new cognitive impairment temporally associated with COVID-19. Third, selection bias cannot be ruled out. For ICU and WARD patients, it is possible that both those doing extremely well and those doing very badly were uninterested in participating in our study. For the HOME group, it is possible that those concerned about their health were keener to participate. For non-COVID controls, the fact that our study included neuropsychological testing might have attracted high-functioning individuals concerned about their memory. However, this is inherent in studies where participation is voluntary. Fourth, this was a single-centre study. However, during the first and second waves of COVID-19, the majority of the disease burden in Finland occurred in the capital region, and it is unlikely that the slightly higher number of patients in a multi-centre design, with the eventual cost of differences in testing, would have improved the overall quality of the study. Lastly, the CONTROL group did not undergo laboratory testing in order to confirm their history of no SARS-CoV-2 infection, and, thus, we cannot rule out a subclinical infection.

## Conclusions

Our study showed that ICU-treated COVID-19 patients, compared to patients with less severe acute COVID-19 or non-COVID controls, showed more severe long-term cognitive impairment. Among those with more than 12 years of education, impairment existed particularly in the domains of attention and, for men, of executive functions.

## Supplementary Information

**Additional file 1**: Supplementary methods

## Data Availability

The datasets analysed during the current study are available from the corresponding author on reasonable request.

## Abbreviations

ARDS: Acute respiratory distress syndrome
BMI: Body mass index
CCI: Charlson comorbidity index
COPD: Chronic obstructive pulmonary disease
COVID-19: Coronavirus disease 2019
GLM: General linear model
ICDSC: Intensive Care Delirium Screening Checklist
ICU: Intensive care unit
IES-6: Impact of Event Scale-6
IMV: Invasive mechanical ventilation
IQCODE: Informant Questionnaire on Cognitive Decline in the Elderly
IQR: Interquartile range
MERS: Middle East respiratory syndrome
mRS: Modified Rankin scale
NS: Non-significant
PaO_2_/FiO_2_: Ratio of arterial oxygen partial pressure to fractional inspired oxygen
PHQ-9: Patient Health Questionnaire 9
SARS: Severe acute respiratory syndrome
SARS-CoV-2: Severe acute respiratory syndrome coronavirus 2
SD: Standard deviation
