# Supplementary material for: Long-term cognitive functioning is impaired in ICU-treated COVID-19 patients: a comprehensive controlled neuropsychological study
Source: Crit Care. 2022 Jul 20;26:223. doi: 10.1186/s13054-022-04092-z (PMC9297673; doi:10.1186/s13054-022-04092-z)
Supplement: Supplementary file 1 — Additional file 1: Supplementary methods [file 13054_2022_4092_MOESM1_ESM.docx]

**Additional file 1: Supplementary methods**

**Long-term cognitive functioning is impaired in ICU-treated COVID-19 patients. A comprehensive controlled neuropsychological study.**

Henriikka Ollila, M.D.^1^ (ORCID id 0000-0002-0833-5644), Riikka Pihlaja, LicPsych^2,3^ (ORCID id 0000-0001-8724-5227), Sanna Koskinen, Ph.D.^2^ (ORCID id 0000-0002-3453-5084), Annamari Tuulio-Henriksson, Ph.D.^2^ (ORCID id 0000-0002-3580-3693), Viljami Salmela, Ph.D.^2^ (ORCID id 0000-0001-7218-5321), Marjaana Tiainen, M.D., Ph.D.^4^ (ORCID id 0000-0001-5107-1990), Laura Hokkanen, Ph.D., professor ^2^ (ORCID id 0000-0001-8342-9248), Johanna Hästbacka, M.D., Ph.D.^1^ (ORCID id 0000-0002-3613-7231)

^1^ Department of Anaesthesiology, Intensive Care and Pain Medicine, Helsinki University Hospital and University of Helsinki, Helsinki, Finland

^2^ Department of Psychology and Logopaedics, Faculty of Medicine, University of Helsinki, Helsinki, Finland

^3^ Division of Neuropsychology, HUS Neurocentre, Helsinki University Hospital and University of Helsinki, Helsinki, Finland

^4^ Department of Neurology, Helsinki University Hospital and University of Helsinki, Helsinki, Finland

Laura Hokkanen and Johanna Hästbacka contributed equally and should both be considered last author.

Corresponding author: Henriikka Ollila, henriikka.ollila@helsinki.fi

**Supplementary methods**

**Definitions of the clinical variables**

Proning: The number of prone positioning treatments occurring during invasive mechanical ventilation (IMV) was counted as proning.

Duration of invasive mechanical ventilation (IMV): Duration of IMV was counted in days including intubation and extubation days. In the case of several IMV episodes, days were summed to get the total number.

Delirium: Intensive care delirium screening checklist (ICDSC) points of four or above determined delirium diagnosis, when available, but, because of the on-going pandemic, patients treated in units, where ICDSC was unavailable, delirium or symptoms of delirium mentioned in the patient chart were eligible for diagnosis [1]. Days, when ICDSC was greater or equal to four points, confirmed the duration of delirium; duration was undefined when the diagnosis was based on patient charts instead of ICDSC points.

**Neuropsychological methods**

We evaluated cognitive functioning in a comprehensive neuropsychological assessment. As registered at ClinicalTrials.gov (NCT04864938), we selected the main outcome variables to represent three domains: attention, executive functions, and memory. We evaluated each domain with three subtests (Table E1) and calculated a total cognitive score as the sum of the domain scores.

To be able to compare subtests measured in different scales, we first standardised the raw scores of the selected nine subtest variables using the mean and standard deviation of the whole study population as a reference. Standardisation was performed by subtracting the reference mean from the individual raw score and then dividing the difference by the reference standard deviation (see equation below). The standardised scores were then summed. In all scores, a higher value indicates better performance.

$$Z=\frac{x- \bar{x}}{S}$$

where $x$ is the individual raw score, $\bar{x}$is the mean of the reference sample, and *S* is the standard deviation of the reference sample.

We imputed single missing values (one case had missing values in the two Stroop variables, two cases had missing values in Continuous Performance Test, one case had missing value in Wechsler Memory Scale logical memory) using the subgroup mean.

We included a non-COVID control group, because no age, sex and education stratified normative cut-off values are available for these tests in Finnish, and the control group allowed controlling for these confounders. Also, the pandemic situation can affect the performance of the population in general, not only of those having had COVID-19, and this can be controlled for with a control group evaluated during the same pandemic.

**Table E1** Neuropsychological subtests included in the calculation of total cognitive score

| Domains and tests | Task description | Analysed variable | Original reference |
| --- | --- | --- | --- |
| **Attention** | | | |
| WAIS-IV Coding | Subject copies symbols that are paired with numbers in a specified order for 120 seconds | Total raw score, correct items | [2] |
| Continuous Performance Test | Computerized test of 7 minutes duration; letters appear one by one on the screen; subject presses space bar for all letters except for the letter X | Correct reactions | [3, 4] |
| Stroop Naming | Subject names colours from a matrix of 5 x 20 items; four different colours in use | Total time in seconds (reversed in standardisation) | [5] |
| **Executive functions** | | | |
| Trail Making B | Subject connects items with a pencil, alternating between sequencing numbers (1-13) and letters of the alphabet (A-L) | Total time in seconds (reversed in standardisation) | [6] |
| Stroop Interference | Subject names the font colour of colour words ignoring the word from a matrix of 5 x 20 items; four different colours in use | Time in seconds (reversed in standardisation) | [5] |
| Frontal Assessment Battery | Subject completes five ^a^ separate tasks exploring conceptualisation, mental flexibility, motor programming, sensitivity to interference, and inhibitory control | Total score | [7] |
| **Memory** | | | |
| WMS-III Word list, delayed recall | A list of 12 words is presented 4 times in the learning phase; in delayed recall phase subject returns the words approximately 30 mins later | Total raw score, correct items | [8] |
| WMS-III Logical memory, delayed recall | Two stories of 25 bits of information each is read to the subject in the learning phase; in delayed recall phase subject returns the stories approximately 30 mins later | Total raw score, correct items from both stories combined | [8] |
| Rey Complex Figure, delayed recall | A complex figure is copied in the learning phase; in delayed recall phase subject draws the figure from memory approximately 30 mins later | Correct items | [9] |

*WAIS-IV* Wechsler Adult Intelligence Scale-IV, *WMS-III* Wechsler Memory Scale version III

^a^ an item from the original six was omitted due to pandemic: prehension behaviour (environmental autonomy) “Do not take my hands” -task was not performed

**Functional and psychological outcomes**

A modified Rankin Scale (mRS) determined a general functional outcome (Table E2) [10]. In the analyses, scores three to four were combined, and no one reported score five symptoms. The Patient Health Questionnaire 9 (PHQ-9) is a validated tool for measuring depression severity (Table E3) utilising nine criteria each scored from 0 (“not at all”) to three (“nearly every day”), and the sum of the nine items is the PHQ-9 score [11]. The Impact of Event Scale 6 questionnaire (IES-6) for post-traumatic stress includes six items (Table E4), each scored from 0 to four (0 equals “not at all”, 1 “a little bit, 2 “moderately”, 3 “quite a bit”, 4 “extremely”), and the mean of the six items is the IES-6 score [12].

**Table E2** The Modified Rankin Scale

| **Score** | **Description** |
| --- | --- |
| 0 | No symptoms at all |
| 1 | No significant disability despite symptoms: able to carry out all usual duties and activities |
| 2 | Slight disability: unable to carry out all previous activities but able to look after own affairs without assistance |
| 3 | Moderate disability: requiring some help, but able to walk without assistance |
| 4 | Moderately severe disability: unable to walk without assistance, and unable to attend own bodily needs without assistance |
| 5 | Severe disability: bedridden, incontinent, and requiring constant nursing care and attention |

**Table E3** The PHQ-9 scores indicating different levels of depression severity

| **PHQ-9 score** | **Level of depression severity** |
| --- | --- |
| 0 – 4 | Minimal |
| 5 – 9 | Mild |
| 10 – 14 | Moderate |
| 15 – 19 | Moderately severe |
| 20 – 27 | Severe |

*PHQ-9* Patient Health Questionnaire 9

**Table E4** The IES-6 questions for assessing post-traumatic stress

| **Question no.** | **Question** |
| --- | --- |
| 1 | I thought about it when I did not mean to |
| 2 | I felt watchful or on-guard |
| 3 | Other things kept making me think about it |
| 4 | I was aware that I still had a lot of feelings about it, but I didn't deal with them |
| 5 | I tried not to think about it |
| 6 | I had trouble concentrating |

*IES-6* Impact of Event Scale 6

**Reference list**

1. Gusmao-Flores D, Salluh JI, Chalhub RA, Quarantini LC. The confusion assessment method for the intensive care unit (CAM-ICU) and intensive care delirium screening checklist (ICDSC) for the diagnosis of delirium: a systematic review and meta-analysis of clinical studies. Crit Care. 2012;16(4):R115.

2. Wechsler D. WAIS-IV: Wechsler Adult Intelligence Scale —Fourth Edition [Finnish version]. Helsinki: Psykologien Kustannus Oy; 2012.

3. Mueller ST, Piper BJ. The Psychology Experiment Building Language (PEBL) and PEBL Test Battery. J Neurosci Methods. 2014;222:250-9.

4. Conners CK, Epstein JN, Angold A, Klaric J. Continuous performance test performance in a normative epidemiological sample. J Abnorm Child Psychol. 2003;31(5):555-62.

5. Stroop JR. Studies of interference in serial verbal reactions. Journal of Experimental Psychology. 1935;18(6):643-62.

6. Reitan RM. The relation of the trail making test to organic brain damage. J Consult Psychol. 1955;19(5):393-4.

7. Dubois B, Slachevsky A, Litvan I, Pillon B. The FAB: a Frontal Assessment Battery at bedside. Neurology. 2000;55(11):1621-6.

8. Wechsler D. WMS-III: Wechsler Memory Scale — Third Edition [Finnish version]. Helsinki: Psykologien Kustannus Oy; 2008.

9. Corwin J, Bylsma FW. Psychological examination of traumatic encephalopathy. Clinical Neuropsychologist. 1993;7(1):3-21.

10. van Swieten JC, Koudstaal PJ, Visser MC, Schouten HJ, van Gijn J. Interobserver agreement for the assessment of handicap in stroke patients. Stroke. 1988;19(5):604-7.

11. Kroenke K, Spitzer RL, Williams JB. The PHQ-9: validity of a brief depression severity measure. J Gen Intern Med. 2001;16(9):606-13.

12. Hosey MM, Leoutsakos JS, Li X, Dinglas VD, Bienvenu OJ, Parker AM, et al. Screening for posttraumatic stress disorder in ARDS survivors: validation of the Impact of Event Scale-6 (IES-6). Crit Care. 2019;23(1):276.
